# Fluctuation of fine motor skills throughout the menstrual cycle in women

**DOI:** 10.1038/s41598-024-65823-6

**Published:** 2024-07-02

**Authors:** Koyuki Ikarashi, Daisuke Sato, Mutsuaki Edama, Tomomi Fujimoto, Genta Ochi, Koya Yamashiro

**Affiliations:** 1 Institute for Human Movement and Medical Sciences, Niigata University of Health and Welfare, 1398 Shimami-Cho, Kita-Ku, Niigata, 950-3198 Japan; 2 Department of Health and Sports, Niigata University of Health and Welfare, Niigata, Japan; 3 Department of Physical Therapy, Niigata University of Health and Welfare, Niigata, Japan

**Keywords:** Menstrual cycle, Fine motor skills, Intracortical inhibition, Sensorimotor integration, Grooved pegboard task, Force modulation task, Motor control, Sensorimotor processing

## Abstract

The effect of the menstrual cycle on fine motor skills is unclear. This study determined whether the menstrual cycle affected fine motor skills and related neural activities. Nineteen women with regular menstrual cycles were tested for fine motor skills using two types of tasks: grooved pegboard task (GPT), which evaluates motor control with high freedom of movements, and force modulation task (FMT), which evaluates more complex and fine motor control with low freedom of movements. We also assessed primary motor cortex intracortical circuits and sensorimotor integration using paired-pulse transcranial magnetic stimulation to reveal why the menstrual cycle affects fine motor skills. The present study indicated that fine motor skills assessed by FMT varied throughout the menstrual cycle while those measured by GPT did not. These results suggest that fine motor skills requiring more complex and fine control may be affected by the menstrual cycle. Additionally, changes in fine motor skills throughout the menstrual cycle may be associated with the severity of menstruation-related symptoms.

## Introduction

Fine motor skills, requiring dexterity and force modulation, play a crucial role in outstanding athletic performance and skill acquisition as well as activities of daily living (including handling utensils, writing, and typing). Currently, there is a debate on whether these skills change during the menstrual cycle. The menstrual cycle entails modulated periodic fluctuations of the levels of sex steroid hormones, including estradiol (E2) and progesterone (P4) and consists of three phases: (1) follicular phase when both E2 and P4 are at low levels, (2) preovulatory phase with a high E2 level and low P4 level, and (3) mid-luteal phase when both E2 and P4 are at high levels. Several studies have reported that fine motor skills improve during the preovulatory^1^ and mid-luteal phases of the menstrual cycle^2,3^, whereas others have reported no changes^4,5^. One of the reasons for these inconsistent results is the tasks that have been used to measure fine motor skills (including Perdue pegboard task and grooved pegboard task [GPT]). These tasks involve high freedom of movements and are affected by multiple factors, including movement velocity and hand–eye coordination^6^. If the menstrual cycle can affect these factors and others that may be involved in motor performance (such as psychological and cognitive aspects), the true impact of the menstrual cycle on fine motor skills may be masked. Accordingly, a task that excludes those factors should be used to clarify the difference in fine motor skills throughout the menstrual cycle. This study used the force modulation task (FMT), which requires complex and fine force control using only the fingertips.

An additional reason for the inconsistent results may be that the measurement phases and their definitions differ across studies. Some previous studies have compared two phases^1,4,7–9^ while others have compared three^2,3,10^, which is based on the sex steroid hormone levels. The definition of the follicular phase of the menstrual cycle is also inconsistent; some studies define the follicular phase as a period during menstruation^10^ and others have define it as periods during and after menstruation^3,8^. However, there is a crucial difference related to the presence or absence of psychological and physical symptoms, although there is no difference in the sex steroid hormone levels during these periods. Various menstruation-related symptoms, including abdominal pain, irritability, and depressed mood, are observed more during than after menstruation. These symptoms affect cognitive and motor functions, behavior, and their associated neural activities^8,11–13^. In other words, the effect of symptoms on fine motor skills and related primary motor cortex (M1) intracortical circuits may differ during and after menstrual periods, and these periods should be distinguished using menstruation-related symptoms. The present study resolved the ambiguity of the definition of the follicular phase as follows: the period during which menstruation-related symptoms are likely to appear is termed as the menstruation phase and period when they are not is termed the follicular phase.

The improvement of fine motor skills involves the intracortical circuits in the M1^14,15^, and it is evaluated by measuring short-interval intracortical inhibition (SICI) and short-interval intracortical facilitation (SICF) using a paired-pulse transcranial magnetic stimulation (TMS) paradigm with short inter-stimulus intervals (ISIs). Previous pharmacological studies have demonstrated that SICI is increased by gamma-aminobutyric acid A (GABA_A_) receptor agonists, including lorazepam and diazepam^16,17^, suggesting that SICI represents GABA_A_ receptor-mediated inhibitory circuits. SICF is related to excitatory interneuron activity summation mediated by glutamate^18^. SICF is decreased by GABA_A_ receptor agonists^19,20^, suggesting that it is controlled by glutamatergic excitation and GABAergic inhibition.

Several studies have indicated that M1 intracortical circuits are affected by the menstrual cycle^3,8,10^, which is attributed to the effects of E2 and P4 on cortical excitability. E2 increases cortical excitability by decreasing GABAergic inhibition and increasing N-methyl-D-aspartate-mediated glutamate receptor activity^21,22^. P4 decreases cortical excitability, enhancing GABA-mediated neurotransmission by binding to GABA_A_ receptors and reducing excitatory glutamate responses^23^. However, the findings on the effect of the menstrual cycle on the M1 intracortical circuits have been contradictory^3,8,10^. Moreover, previous studies have reported that sensorimotor integration, the interaction between the sensory and motor systems, is also involved in fine motor skills^24,25^. Sensorimotor integration can be assessed using short-latency afferent inhibition (SAI) measured by combining single TMS pulses with prior peripheral electrical nerve stimuli at specific ISIs. SAI reflects an inhibitory circuit via GABAergic neuron projecting to M1 pyramidal cells by afferent input^26^. The mechanism of SAI is different from that of SICI and SICF^26^. However, it involves GABAergic neuron-mediated inhibitory circuits, which may change during the preovulatory and mid-luteal phases when E2 and P4 levels fluctuate.

This study aimed to determine whether the menstrual cycle affects fine motor skills using a task that requires more complex and fine motor control with low freedom of movement. We also determined whether intracortical circuits and sensorimotor integration in the M1 are involved in menstrual cycle-induced changes in fine motor skills. In the present study, participants performed the experiments during all four menstrual cycle phases to achieve our objectives. Previous studies have reported that E2 increases cortical excitability by decreasing GABAergic inhibition^21,22^, although M1 intracortical excitability and corticospinal excitability do not change during the follicular and preovulatory phases^3,8,27^. Sensorimotor integration, rather than M1 intracortical circuits, may play a role in the variations observed in fine motor skills across the menstrual cycle. Therefore, we hypothesized that fine motor skills improve during the preovulatory phase relative to the follicular phase. E2 levels are the most elevated and may improve fine motor skills by decreasing GABA_A_ receptor-mediated inhibition assessed by SAI.

## Results

The participants completed the experiment during all four menstrual cycle phases: menstruation, follicular, preovulatory, and mid-luteal phases. Sex steroid hormone levels, fine motor skills, M1 intracortical circuits, sensorimotor integration, and menstruation-related symptoms were measured for each phase (see Methods for details).

### Salivary sex steroid hormone levels

**Figure 1:**
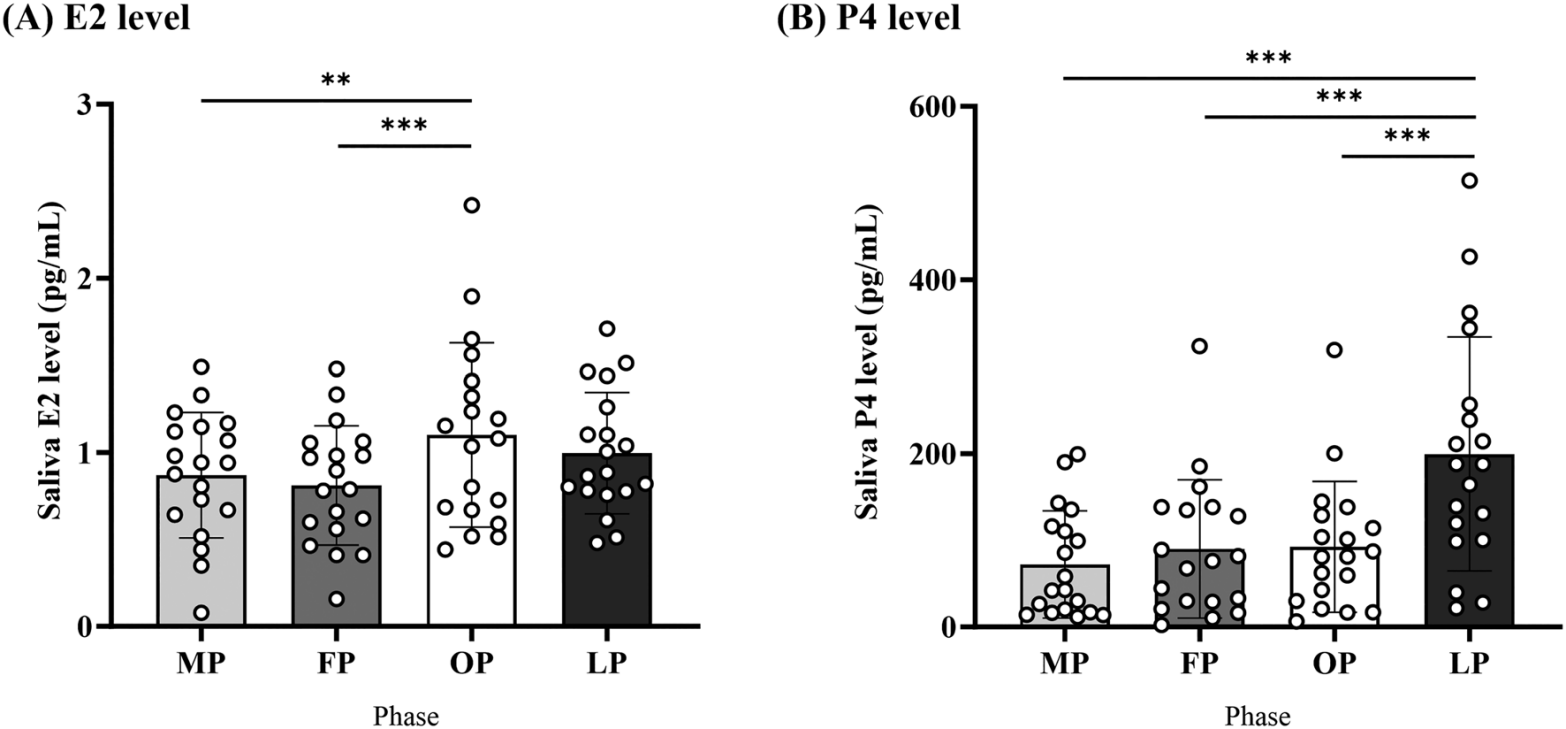
Salivary sex steroid hormone levels. (**A**) E2 levels were higher for the OP than for the FP and MP. (**B**) P4 levels increased for the LP than during the other phases. *E2* estradiol, *P4* progesterone, *MP* menstruation phase, *FP* follicular phase, *OP* preovulatory phase, *LP* mid-luteal phase.

Figure 1A,B show differences in the E2 and P4 levels throughout the menstrual cycle. The E2 levels were significantly higher during the preovulatory phase than during the menstruation and follicular phases (both *b* > 0.81, both *SEb* = 0.19, both *|t|*> 2.65, both *P* < 0.01). The P4 levels were significantly higher during the mid-luteal phase than during the other phases (all *b* > 100.20, all *SEb* = 39.77, all *|t|*> 5.17, all *P* < 0.001).

### Psychological data

**Figure 2:**
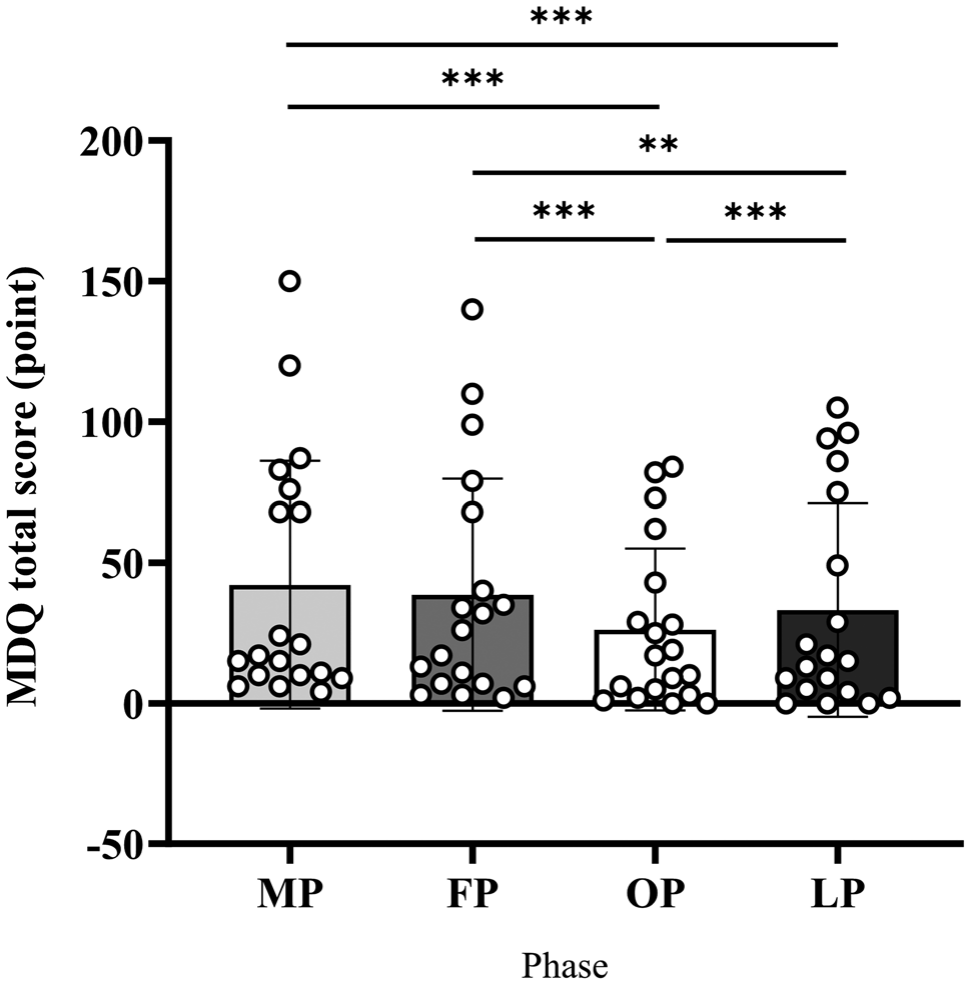
MDQ total score. MDQ total score for each menstrual cycle phase. The MDQ total score was lower during the OP and LP than during the MP and FP. *MDQ* menstrual distress questionnaire; *MP* menstruation phase, *FP* follicular phase, *OP* preovulatory phase, *LP* mid-luteal phase.

The present study used the menstrual distress questionnaire (MDQ) to assess the severity of menstruation-related symptoms. Figure 2 shows the total MDQ score for each phase. Higher total MDQ scores indicate more severe menstruation-related symptoms. The total MDQ score was significantly higher during the menstruation, follicular, and mid-luteal phases (all *b* > 2.94, all *SEb* > 0.32, all *|t|*> 3.90, all *P* < 0.001) than during the preovulatory phase. The MDQ total scores during the menstruation and follicular phases were significantly higher than those during mid-luteal phase (both *b* > 2.94, both *SEb* > 0.31, both *|t|*> 2.79, both *P* < 0.05).

### Behavioral data

**Figure 3:**
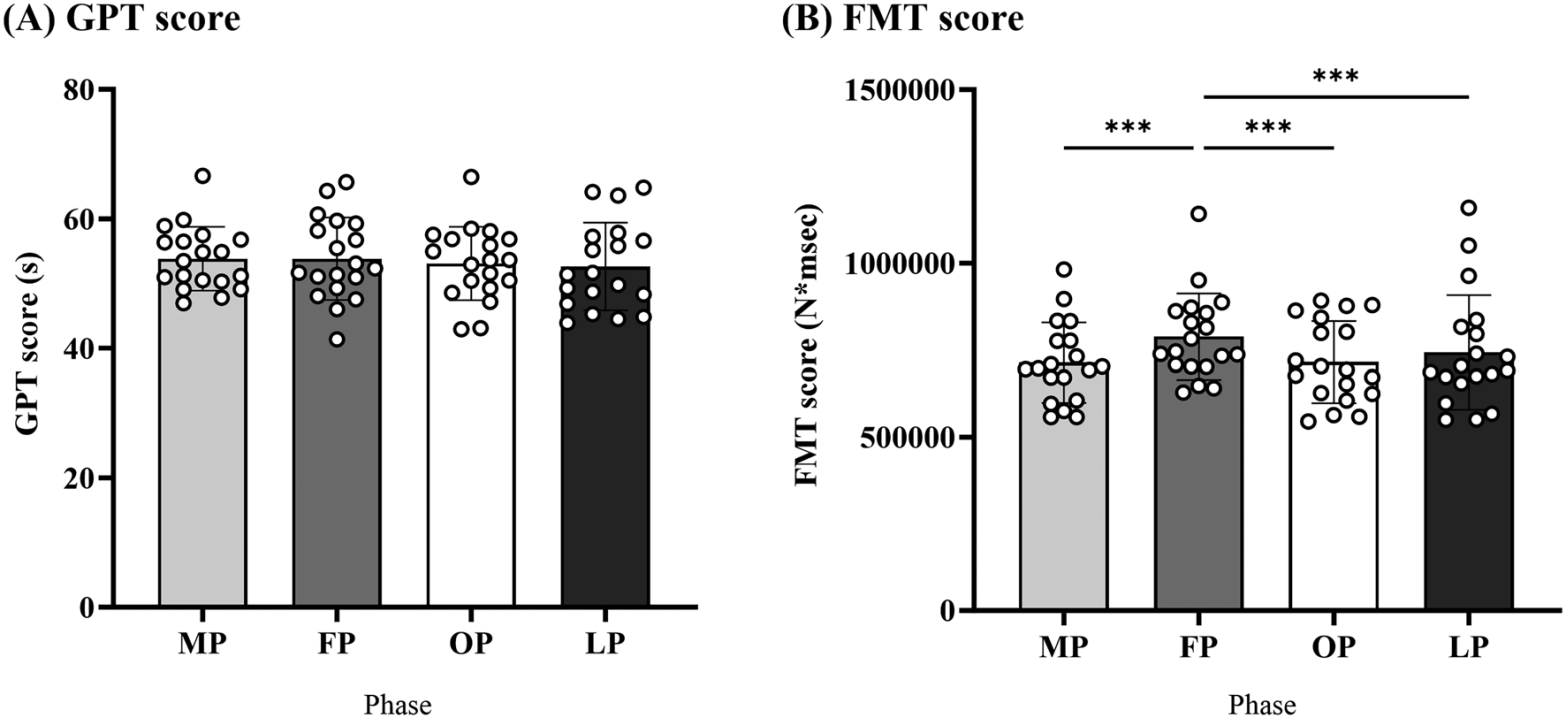
Fine motor skills during the menstrual cycle. (**A**,**B**) Show the GPT and FMT scores for each menstrual cycle phase. There were no differences in the GPT score across the menstrual cycle, whereas the FMT score was significantly worse during the FP than during all other menstrual phases. *GPT* grooved pegboard test, *FMT* force modulation task, *MP* menstruation phase, *FP* follicular phase, *OP* preovulatory phase, *LP* mid-luteal phase.

**Figure 4:**
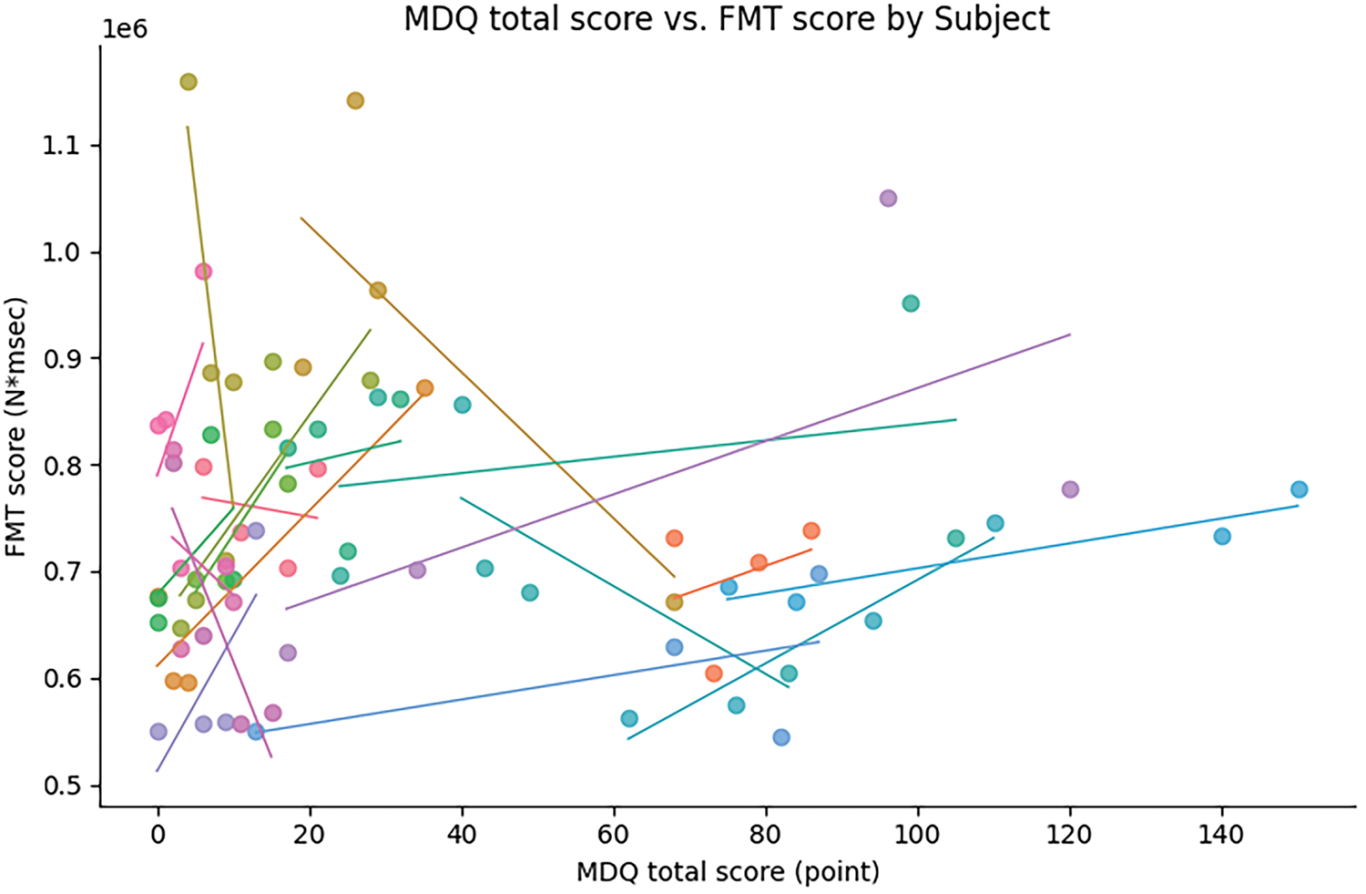
Relationship between FMT score and MDQ score. Repeated measures correlation found a positive correlation trend between FMT score and MDQ score. *FMT* force modulation task, *MDQ* menstrual distress questionnaire.

Fine motor skills were measured using GPT and FMT during each menstrual cycle phase. The GPT score did not change throughout the menstrual cycle (all *b* > 52.61, all *SEb* > 2.33, all *|t|*< 1.144, all *P* > 0.05, Fig. 3A). The FMT score was significantly higher for the follicular phase than for the other menstrual cycle phases (all *b* < 13.47, all *Seb* > 0.22, all *|t|*> 265.09, all *P* < 0.001), indicating that fine motor skills requiring complex and fine control are impaired during the follicular phase (Fig. 3B). This change was related to the total MDQ score (*b* = 13.45, *Seb* = 0.22, *z* = 225.92, *P* < 0.001). The FMT and MDQ scores were positively correlated (r = 0.22, 95% CI [− 0.03, 0.46], *P* = 0.08, Fig. 4), suggesting that more severe menstrual-related symptoms are associated with reduced fine motor skills.

### Neurophysiological data

**Figure 5:**
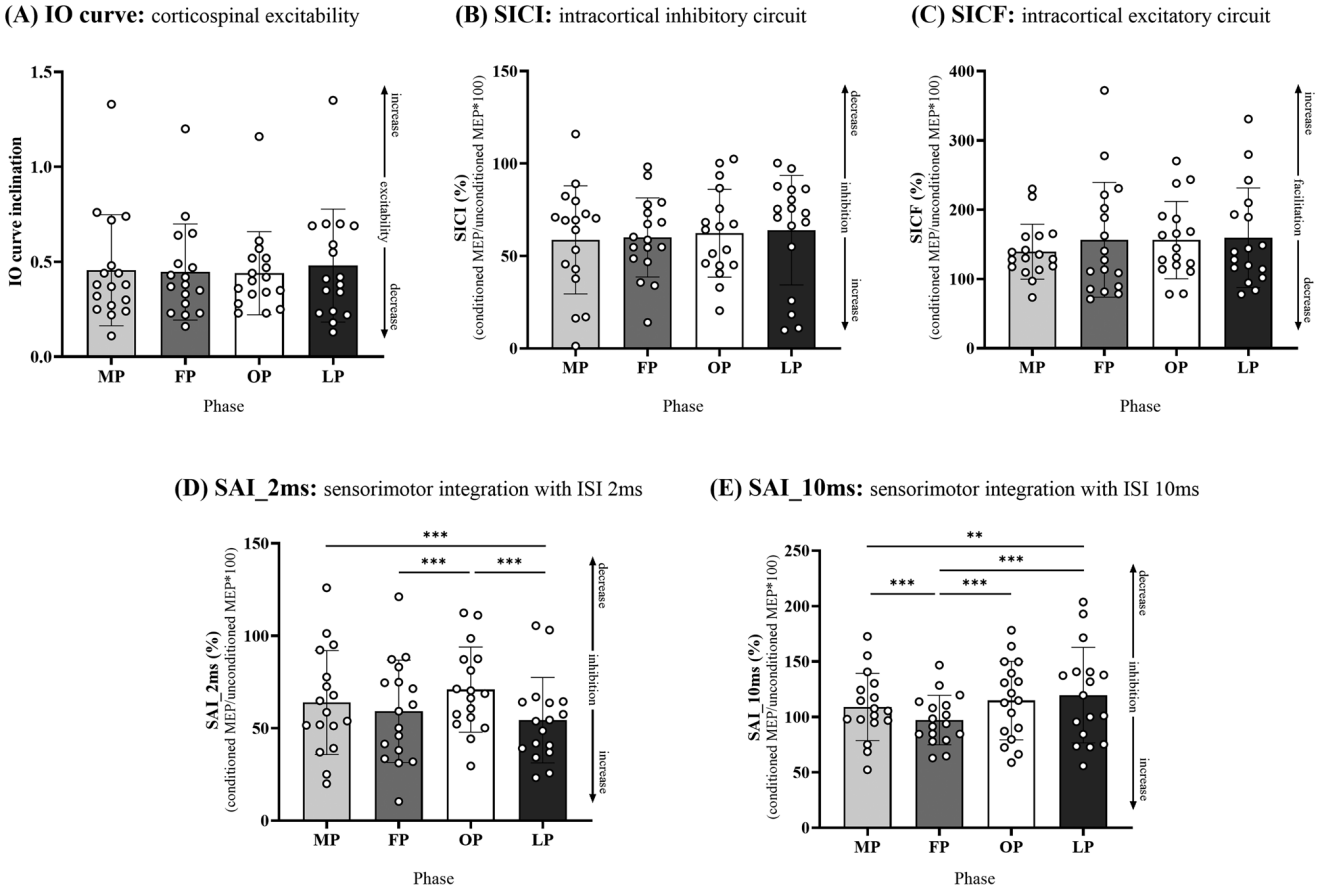
Intracortical circuits in M1. (**A**–**C**) Changes in corticospinal excitability and M1 intracortical inhibition during each menstrual cycle phase. IO curve inclination, SICI, and SICF did not change across the menstrual cycle. (**D**) and (**E**) indicate sensorimotor integration during each menstrual cycle. SAI_2ms decreased during the OP relative to the FP and LP. SAI_10ms decreased during the MP, OP, and LP than during the FP. *SICI* short-interval intracortical inhibition, *SICF* short-interval intracortical facilitation; *SAI* short-latency afferent inhibition, *ISI* inter-stimulus interval, *MP* menstruation phase, *FP* follicular phase, *OP* preovulatory phase, *LP* mid-luteal phase.

Figures 5A–E show M1 intracortical circuits and sensorimotor integration. Input–output (IO) curve inclination, SICI, and SICF were not significant throughout the menstrual cycle phase (IO curve inclination: all *b* > − 0.87, all *SEb* > 0.94, all |*t*|> 0.15, all *P* > 0.05; SICI: all *b* > 2.72, all *SEb* = 10.65, all |*t*|> 0.85, all *P* > 0.05; SICF: all *b* > 139.38., all *SEb* > 25.26, all |*t*|> 1.63, all *P* > 0.05). This suggests that corticospinal excitability and M1 intracortical circuits may not be affected by the menstrual cycle. Our results indicate that sensorimotor integration changes throughout the menstrual cycle. SAI_2ms was significantly decreased during the preovulatory phase relative to the follicular and mid-luteal phases (both *b* > 54.10, both *SEb* > 4.63, both *|t|*> 4.32, both *P* < 0.001) and during the menstruation phase relative to the mid-luteal phase (*b* = 63.65, *SEb* = 4.43, *t* = 3.62, *P* < 0.001). SAI_10ms was significantly decreased during the menstruation, preovulatory, and mid-luteal phases relative to the follicular phase (all *b* > 108.83, all *SEb* > 5.88, all *t* > 3.40, all *P* < 0.05) and during the mid-luteal phase relative to the menstruation phase (*b* = 108.83, *SEb* = 6.32, *t* = − 2.88, *P* < 0.01). However, these change in neurophysiological data (i.e., SAI_2ms and _10ms) were not related to the MDQ score (SAI_2ms: *b* = 62.01, *Seb* = 1.35., *z* = − 0.17, *P* = 0.87; SAI_10ms: *b* = 4.66, *Seb* = 0.24, *z* = 0.46, *P* = 0.65). No significant correlations of the FMT score with the SAI_2ms and _10ms were found (SAI_2ms: r = 0.06, 95% CI [− 0.22, 0.33], *P* = 0.68; SAI_10ms: r = 0.06, 95% CI [− 0.21, 0.33], *P* = 0.66).

All raw data are presented in Supplementary Tables S1–5.

## Discussion

The present study aimed to reveal whether the menstrual cycle altered fine motor skills and associated M1 intracortical circuits and sensorimotor integration. We found that the menstrual cycle modulated more complex and fine motor skills assessed using FMT, which are explained by the change in menstruation-related symptoms and sensorimotor integration.

### Task-dependent change in fine motor skills throughout the menstrual cycle

The present study found that fine motor skills assessed using FMT changed throughout the menstrual cycle, while no change was observed when using the GPT. These results indicate that more complex and fine motor control with low freedom of movement alters throughout the menstrual cycle, which may be attributed to the characteristics of the implemented tasks. The GPT has a high freedom of movement and consists of multiple factors, including movement velocity and hand–eye coordination^6^, which may mask the influence of the menstrual cycle. Several studies have not shown an effect of the menstrual cycle on fine motor skills assessed using the GPT^4,5^. Functional cerebral asymmetries associated with fine motor skills show greater variations throughout the menstrual cycle for complex motor tasks than for simple tasks^28^. Therefore, the menstrual cycle affects more complex and fine motor control with low freedom of movement, and the FMT may be more suitable than the GPT for assessing this effect.

### Effect of menstruation-related symptoms on fine motor skills

The changes in fine motor skills assessed using the FMT throughout the menstrual cycle could be explained by the severity of menstruation-related symptoms. It has been reported that 50–90% of women experience some psychological or physical symptoms, such as mood lability, anxiety, irritability, anger, lower abdominal pain, and headache, during the menstruation and mid-luteal phases^29,30^, and these symptoms affect cognitive and motor performance^11,12^. Our previous study also showed that menstruation-related symptoms may be related to impaired motor learning during the mid-luteal phase^31^. The present study revealed that the fine motor skills were impaired during the follicular phase and that the menstruation-related symptoms were worse during this phase than during the preovulatory and mid-luteal phases. We found a positive correlation trend between these parameters, indicating that more severe menstruation-related symptoms were associated with reduced fine motor skills. On the other hand, there were no significant correlations between the fine motor skills and sex steroid hormone levels (Supplementary Materials Tables S5 and S6, Figs. S1 and S2). Based on these findings, fine motor skills may be influenced by menstruation-related symptoms rather than the fluctuations of E2 and P4 levels throughout the menstrual cycle. However, there was no difference in the severity of these symptoms during these phases even though this study categorized the menstrual cycle phases based on the menstruation-related symptoms (i.e., menstruation and follicular phases). Thus, factors other than menstruation-related symptoms may be associated with the differences in fine motor skills during the menstruation and follicular phases.

### Mechanisms of changes in fine motor skills throughout the menstrual cycle

We speculate that the changes in the fine motor skills throughout the menstrual cycle may be related to sensorimotor integration. The present study revealed that fine motor skills were superior during the preovulatory phase relative to the follicular phase. This may be associated with modulated sensorimotor integration via GABAergic neuron-mediated inhibitory circuits, as indicated by the decrease in SAI_2ms. Di Lazzaro et al.^26^ suggested that SAI may be caused by the enhancement of GABAergic neurons projecting to M1 pyramidal cells by afferent input. The present study showed that SAI_2ms was decreased and the E2 levels were higher during the preovulatory phase than during the follicular phase. Since E2 decreases GABAergic inhibition^21–23^, the decreased SAI_2ms during the preovulatory phase may be attributed to decreased GABAergic inhibition associated with elevated E2 levels. Some studies reported that the decreases in GABA_A_ receptor-mediated inhibition are plastic changes associated with increased motor skills^32^. A recent review has shown that sensorimotor integration assessed by SAI is related to improved motor skills^33^. A previous electroencephalography (EEG) study reported that the activities of several cortical and subcortical regions involved in sensorimotor integration are significantly higher during the preovulatory phase^34^. Therefore, the differences between the fine motor skills during the follicular and preovulatory phases may be explained by changes in sensorimotor integration via GABAergic neuron-mediated inhibitory circuits.

We also showed unexpected results that fine motor skills were superior not only during the ovulatory phase but also during the menstruation and mid-luteal phases relative to the follicular phase. These results may be explained by the decrease in SAI_10ms during those phases. We speculate that some modulation of sensorimotor processing via basal ganglia may be related to superior fine motor skills. Kessler et al.^35^ reported that only SAI_10ms decreases in patients with focal hand dystonia with no changes in other SAIs with several ISIs. Dystonia, including focal hand dystonia, is generally considered a dysfunction of the basal ganglia. Several studies have observed abnormal SAI in patients with motor deficits. A previous study reported that the interaction between the sensory and motor systems involves a complex brain network, including the M1, primary somatosensory cortex, basal ganglia, and cerebellum^36^. Given these findings, SAI_10ms may reflect an inhibitory circuit involving the basal ganglia-thalamic-cortical regions different from SAI_2ms although the detailed mechanisms of SAI_10ms remain unclear. Furthermore, a functional magnetic resonance imaging study reported the functional and structural changes in the basal ganglia throughout the menstrual cycle^37,38^. While this is speculative, these changes in the basal ganglia throughout the menstrual cycle may affect an inhibitory circuit involving basal ganglia-thalamic-cortical regions. This may result in improved fine motor skills during the menstruation, preovulatory, and mid-luteal phases. However, further research is needed because the detailed mechanisms of SAI_10ms remain unclear.

### Implementation and limitations of the present study

This study is the first to reveal changes in fine motor skills throughout the four menstrual cycle phases and indicate that these changes may be modulated by sensorimotor integration and menstruation-related symptoms. In our study, superior FMT scores were observed during the menstruation, preovulatory, and mid-luteal phases relative to the follicular phase, indicating that motor skills requiring fine force modulation are enhanced during these phases. Fine motor skills represent a critical component for achieving superior performance during sports activities^39^. Building on the present result and our previous work^31^, which demonstrated that motor learning requiring force modulation is enhanced during the preovulatory phase relative to the mid-luteal phase, the preovulatory phase may be the most suitable phase for achieving high motor performance requiring fine force modulation and associated learning. However, our findings are based on a limited sample of 19 participants from the general population. It is unclear whether the menstrual cycle also affects fine motor skills, M1 intracortical circuits, and sensorimotor integration in athletes. Additionally, fine motor skills may involve subcortical regions such as the hippocampus and basal ganglia in addition to cortical regions, although we did not verify this. The hippocampus interacts with the motor system and plays a role in motor learning and volitional finger movements^40^. It has also been reported that the hippocampal structure and function fluctuate throughout the menstrual cycle^41^, and the possibility that these changes in the hippocampus affect the present results cannot be ruled out. Therefore, further research should involve larger samples that include highly skilled athletes and evaluate the neural activities of regions related to sensorimotor performance and control, including the hippocampus and basal ganglia.

## Conclusion

Fine motor skills requiring more complex and fine force modulation with lower freedom of movement are affected by the menstrual cycle. This may be attributed to the severity of menstruation-related symptoms and changes in sensorimotor integration throughout the menstrual cycle.

## Methods

### Participants

The sample size was calculated and determined using Superpower^42^, which indicated that a sample of 17 for each menstrual cycle phase would be sufficient for an 85% power and an effect size of 0.4. Twenty-four healthy, right-handed women aged 20–24 years were recruited for this study. The participants were required to have no history of neurological or psychiatric disorders (including premenstrual dysphoric disorder) or use of hormonal contraceptives or other hormonal medications. This study was approved by the ethics committee of the Niigata University of Health and Welfare, Japan (18345-200122). This study conformed to the Declaration of Helsinki and was performed after obtaining written informed consent from the participants.

### Procedure

After recruitment, all participants were instructed to measure their basal body temperature using a thermometer (TOPPAN FORMS, Tokyo, Japan) 2 months before the study to accurately estimate their menstrual cycle length. To estimate the ovulation and E2 elevation days, they were also instructed to use an ovulation test kit (Doctor’s Choice One Step Ovulation Test Clear; Beauty and Health Research, Torrance, CA) from the day after the end of menstruation. We asked the participants to report their basal body temperature, ovulation test results, and onset and end of menstruation using Google Forms. During this period, five participants were excluded from the experiment and analysis: two began using hormonal contraceptives and three had a schedule conflict. The final analysis included 19 participants who had regular menstrual cycles. For behavioral data analysis, we used data from 19 participants. For the TMS data analysis, we used the data of 17 participants, as more than 100% of maximal stimulus output in the TMS was required to induce a motor-evoked potential (MEP) amplitude of 1 mV in two participants.

**Figure 6:**
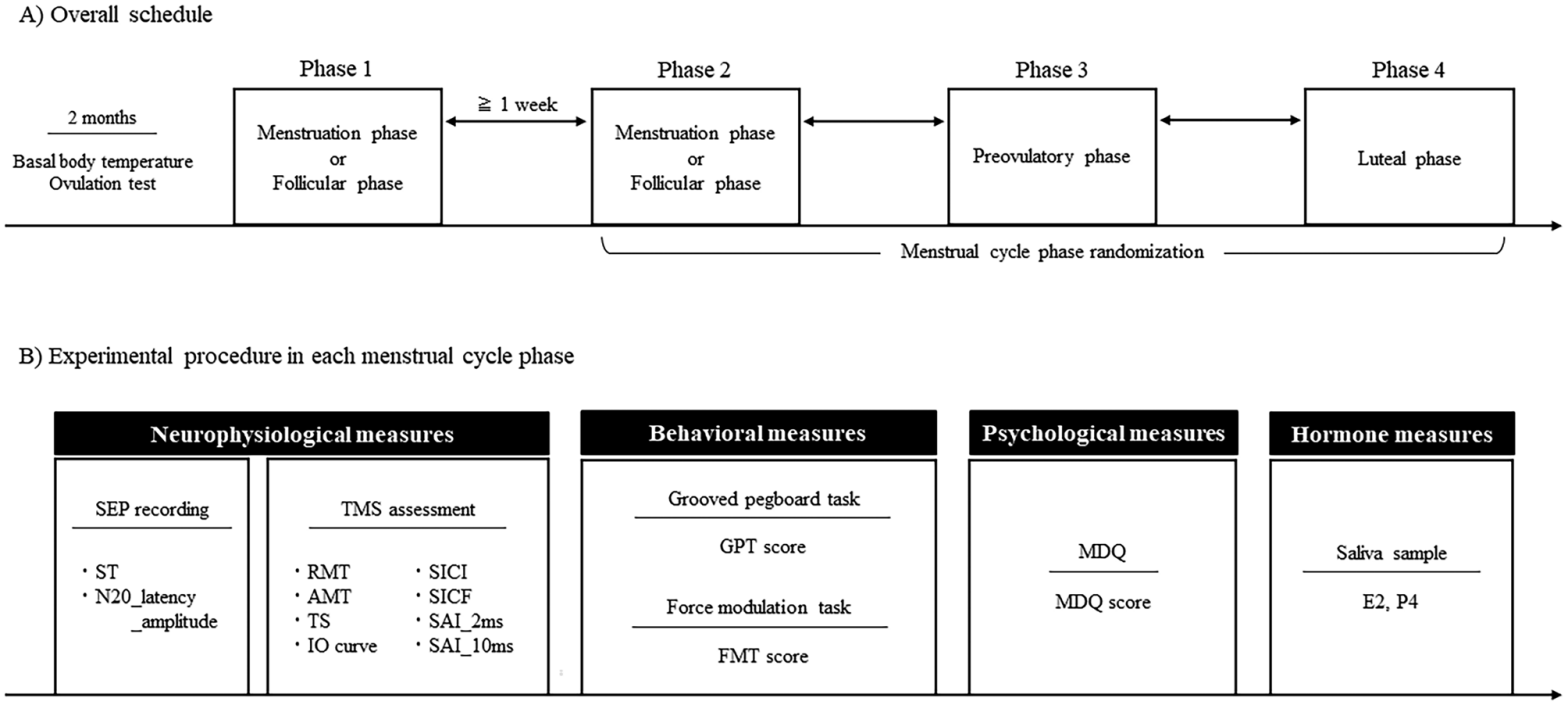
Experimental procedure. Participants started the experiment from the menstruation or follicular phases and completed it for all four menstrual cycle phases: menstruation, follicular, preovulatory and mid-luteal phases. The order of assessment for the phases 2–4 was randomized with an interval of at least one week (**A**). Neurophysiological, behavioral, psychological, and sex steroid hormone assessments performed during each menstrual cycle phase (**B**). *SEP* somatosensory-evoked potential, *ST* sensory threshold, *TMS* transcranial magnetic stimulation, *RMT* resting motor threshold, *AMT* active motor threshold, *TS* test stimulation, *SICI* short-interval intracortical inhibition, *SICF* short-interval intracortical facilitation, *SAI* short-latency afferent inhibition, *GPT* grooved pegboard test, *FMT* force modulation task, *MDQ* menstrual distress questionnaire, *E2* estradiol, *P4* progesterone.

The experimental procedure is shown in Fig. 6. The participants were assessed during all four menstrual cycle phases (menstruation, follicular, preovulatory, and mid-luteal phases). These phases were defined based on the sex steroid hormone levels, as well as the menstruation-related symptoms (see Introduction section). The menstruation phase presenting with menstruation-related symptoms was defined as 1–4 days after the onset of menstruation; the follicular phase, without menstruation-related symptoms, was defined as 1–4 days after the end of menstruation; the preovulatory phase was defined as 2–3 days after the date of a positive ovulation test; and the mid-luteal phase was defined as between 3 days after the ovulation and 3 days before the onset of the next menstruation. Ovulation was verified using the ovulation test and defined as the change of the positive result (luteinizing hormone level above the baseline) to the negative result (luteinizing hormone level below the baseline). The onset of the next menstruation for each participant was predicted based on the menstrual cycles for the previous 2 months.

The order of assessment of the phases was randomized for the participants to prevent sequence and learning effects. Participants started the experiment from the menstruation or follicular phases based on the previous results about learning effects of sensorimotor skills^31^. The order of subsequent assessment of the phases was randomized for the participants with an interval of at least one week. Four participants started the experiment during the preovulatory and mid-luteal phases due to scheduling conflicts. To account for circadian fluctuations, assessments for each phase were performed between 9 AM and 1 PM.

Supplementary Table S1 shows the participant characteristics and number of days characterizing each menstrual cycle phase.

### Sex steroid hormones

#### Saliva sample

After the experiment was completed for each phase, saliva was collected to measure the E2 and P4 levels using a saliva collection kit (SalivaBio A; Salimetrics, Carlsbad, CA). The participants were prohibited from the following before collection: (1) food intake within 60 min; (2) alcohol intake within 12 h; (3) sugary, acidic, or caffeinated beverage intake; (4) dairy product intake within 20 min; (5) tooth brushing within 45 min; and (6) dental treatment within 48 h. The saliva of the participants was collected by ejecting it into a container (Cryovial; Salimetrics) using a straw (Saliva Collection Aid; Salimetrics). The collected saliva samples were immediately stored in a freezer at − 80 °C. After all samples were collected, the E2 and P4 levels were analyzed by Funakoshi Corporation (Tokyo, Japan). The E2 and P4 levels were measured using high-sensitivity salivary immunoassay kits (17β-Estradiol Enzyme Immunoassay kit and salivary progesterone Enzyme Immunoassay kits; Salimetrics). The samples were thawed at room temperature, mixed by vortexing, centrifuged at 1500×*g* for 15 min, and analyzed using enzyme-linked immunosorbent assay. The dilution factor was uniformly onefold (undiluted).

### Psychological assessment

#### Menstrual distress questionnaire

To assess the severity of psychological and physical symptoms with the menstrual cycle, the MDQ, which consists of 48 symptoms in eight categories, was administered during each phase^43^. Participants were asked to assess all symptoms with a score of 1 (no symptoms), 2 (minimal), 3 (mild), 4 (moderate), 5 (strong), or 6 (severe). The total score was calculated.

### Behavioral assessments

#### Grooved pegboard task (GPT)

Fine motor skills were assessed using the GPT (Lafayette Instruments, Lafayette, IN)^44^. The GPT requires participants to place key-shaped pegs into a 5 × 5 grid of key-shaped holes with varying orientations. This requires significant visual input including sensory feedback and intention adjustment^45^. The participants used only their right hand and filled the grooved pegboard from left to right and from top to bottom as quickly as possible. Each participant was familiarized with the test by immediately inserting pegs into a single row preceding the timed trial referenced by Feeney et al.^46^. The GPT score corresponds to the duration from the verbal cue to the start of the test to the final peg insertion. Each participant performed the test two times, and a better GPT score was used as a measure of fine motor skill.

#### Force modulation task (FMT)

Finer motor skills were assessed using an FMT with a custom-built PC program (DASYLab v2016; Digilent, Pullman, WA)^31^. Before performing the FMT, the maximal voluntary contraction (MVC) force in the right first dorsal interosseous (FDI) of the participants was measured. The participants were required to gradually increase the force over 3 s and maintain the maximal force for approximately 3 s. This was repeated, and a larger force was used as the MVC. Subsequently, the participants performed the FMT. For this, the participants had to adjust a red line to a target black line on a screen by pinching and controlling a force transducer in their thumb and index fingers. The red and target black lines simultaneously appeared and moved from the left side to the right side of the screen. The red line moved upward and downward when the pinch force was increased and decreased, respectively. The force modulation range was set as 5–15% of the MVC of the participants. All participants performed two sets, with each comprising 10 trials. The deviation area calculated for each set was averaged and defined as the FMT score.

### Neurophysiological assessments

#### Somatosensory-evoked potential recording and analysis

EEG signals were recorded at a sampling rate of 5 kHz using a digital EEG amplifier (ActiCHamp; Brain Products GmbH, Gilching, Germany) and multifunctional recording software (Brain Vision Recorder, Brain Products GmbH). The EEG signals were recorded using scalp Ag/AgCl surface electrodes. According to the international 10–20 system of EEG electrode placement, somatosensory-evoked potentials (SEPs) were recorded using active electrodes placed at C3’ (located 2 cm posterior to C3). A reference electrode was placed at Fz, with the ground electrode at AFz^47^.

The right median nerve was stimulated at the wrist with a constant-current stimulator (SEN-7203; Nihon Kohden, Tokyo, Japan), with the anode placed on the wrist crease and the cathode at 2 cm proximally. Monophasic square-wave pulses of 200-µs duration were delivered at 250% of the sensory threshold (ST). Single-pulse stimuli were applied at a frequency of 2 Hz for 300 stimulation trials controlled using a pulse control system (Pulse Timer II; Medical Try System, Tokyo, Japan).

Offline analysis was conducted using an analysis software (Brain Vision Analyzer 2, Brain Products GmbH). Continuous data were band-pass filtered from 3 Hz to 2 kHz^48^ and segmented, beginning 20 ms before each initial stimulus in each sweep and lasting for 100 ms, with baseline correction for 20 ms before the initial stimulus. Segmented epochs with responses exceeding ± 70 μV were rejected from the analysis, and the remaining data were averaged. The N20 latency and amplitude were analyzed at C3’.

#### TMS assessment and electromyographic recording

TMS was used to assess the M1 intracortical circuits. A figure-eight coil (D70 Alpha Flat Coil [4170-00]; Magstim, Whitland, UK) was connected to two Magstim 200 stimulators and held over the optimum position at the left M1 (i.e., hotspot) to elicit MEPs in the right FDI muscle. The coil was held tangentially on the scalp at an angle of 45° to the midsagittal plane to induce a posterior-anterior current across the central sulcus^49^. A coil navigation system (Brainsight; Rogue Resolution, Cardiff, UK) was used to maintain the coil position in three-dimensional space relative to the head.

The resting motor threshold (RMT) was the lowest TMS intensity required to elicit an MEP with a peak-to-peak amplitude > 50 µV in 5 out of 10 consecutive stimulations while the participants were at rest^50^. The active motor threshold (AMT) was defined as the lowest intensity required to evoke an MEP of ≥ 200 μV in at least 5 out of 10 consecutive trials while maintaining a 5–10% MVC of the FDI^51^. Muscle contraction was monitored visually using a digital oscilloscope, with the participants being able to monitor and adjust muscle contraction to maintain the required MVC of 5–10%.

Surface electromyography recordings were acquired in a belly-to-tendon montage from the FDI and right-hand abductor pollicis brevis muscles. Signals were amplified with a gain of 1000 (BA2008; Miyuki Giken, Tokyo, Japan), band-pass filtered (5–2000 Hz), digitized at 5 kHz with a 16-bit analog-to-digital converter (Micro 1401-4; Cambridge Electronic Design, Cambridge, UK), and analyzed using data acquisition software (Signal v7, Cambridge Electronic Design) for online monitoring, storage, and offline analysis.

#### Corticospinal excitability

We used an IO curve to evaluate corticospinal excitability by measuring the MEP amplitude produced by single-pulse TMS at six intensities. The TMS intensities ranged from 100 to 150% of the RMT in 10% steps, as determined for each participant. The IO curve was obtained at rest. Ten pulses were delivered for each stimulus intensity, with an ISI of 4–5 s. The stimulus intensities were randomly administered. We excluded the first MEP for each trial from the analysis to prevent collecting the startle and reflex responses.

#### Intracortical excitability

We measured the SICI and SICF at rest to assess the intracortical inhibitory and excitatory circuits involved in the indirect wave 3 (I3 wave) through the GABA_A_ receptor^52^. SICI was studied using the techniques of Kujirai et al.^53^. Paired-pulse TMS was administered through the same stimulating coil over the left motor cortex, and the effect of conditioning stimulus (CS) on test stimulus (TS) was measured. To measure SICI, the CS intensity was set at 90% of the AMT and applied before TS with a 3-ms ISI^54^. For SICF, the CS intensity was set at 90% of the RMT and applied 3 ms after the TS. We measured SAI at rest to assess sensorimotor integration related to the I3 wave through cholinergic and GABAergic neural activities^55,56^. We assessed two types of SAI with an ISI of the latency of the N20 component plus 2 and 10 ms between the CS and TS, termed SAI_2ms and SAI_10ms, respectively. The N20 latency was determined for each participant based on the SEP data. The CS was the median nerve electrical stimulation at 2.5 times the ST intensity. The TS intensity was set to elicit an unconditioned MEP in the relaxed right FDI with approximately 1 mV (0.9–1.1 mV) peak-to-peak amplitude for all stimulus paradigms. The measurement block consisted of 50 trials, including 10 trials for the paradigm of each stimulus (SICI, SICF, SAI_2ms, SAI_10ms, and TS alone). Ten pulses were delivered for the paradigm of each stimulus with an ISI of 4–5 s; stimulus paradigms were randomly administered. SICI, SICF, SAI_2ms, and SAI_10ms were calculated as the ratios of the conditioned MEP amplitude to the unconditioned MEP amplitude. Lower values indicated increased inhibition, while larger values indicated decreased inhibition.

### Statistical analyses

Statistical analyses were conducted in R 4.2.2 (R Foundation, Vienna, Austria) using the “glmer” function of the lme4 package^57^. The dependent variables were E2 and P4 levels, GPT, FMT, IO curve inclination, SICI, SICF, SAI_2ms, SAI_10ms, and MDQ total score. During the first step, our main interest was in the effect of menstrual cycle phases. Accordingly, generalized linear mixed effects models were fitted to the data using the cycle phase as the fixed effects and participant number as a random effect. The present study used the t-statistic and z-statistic when the distribution of each parameter fit a model with a Gaussian or Poisson distribution, respectively. Correlations between behavioral data and neurophysiological and psychological data were examined using repeated measures correlation (R package rmcorr). The rmcorr correlation coefficient determines the common intraindividual relation for paired measurements assessed on two or more occasions for multiple individuals^58^. The *P*-values were FDR-corrected for multiple comparisons, and values of < 0.05 denoted statistical significance.

### Supplementary Information

Supplementary Information.

## Data Availability

The datasets used and/or analyzed during the current study are available from the corresponding author upon reasonable request.
