# Supplementary material for: Fluctuation of fine motor skills throughout the menstrual cycle in women
Source: Sci Rep. 2024 Jul 2;14:15079. doi: 10.1038/s41598-024-65823-6 (PMC11219923; doi:10.1038/s41598-024-65823-6)
Supplement: Supplementary file 1 — Supplementary Information. [file 41598_2024_65823_MOESM1_ESM.docx]

**Supplementary Material**

Table S1 shows participant characteristics and number of days in each menstrual cycle phase.

Tables S2 and S3 show raw psychological and neurophysiological data, respectively, throughout the menstrual cycle.

Table S4 shows raw data of statistical analysis using glmer. Repeated measures correlation was used to evaluate the relationship between behavioral and neurophysiological data and ovarian hormone levels (E2 and P4). The results showed that ovarian hormone levels were not significantly association with behavioral and neurophysiological data (Table S5-6, Figure S1-2). Therefore, it was suggested that ovarian hormone levels may not be a factor that alters fine motor skills or M1 intracortical inhibition and sensorimotor integration.

Table S1. Participants characteristics in the present study

| Participants characteristics | | | | |
| --- | --- | --- | --- | --- |
| Age | 21.73 | ± | 0.93 | years |
| Menstrual cycle length | 30.95 | ± | 3.62 | days |
| Bleeding days | 6.89 | ± | 0.85 | days |
| Day of the data collection in each menstrual cycle phase | | | | |
| Menstruation phase | 3.21 | ± | 0.83 | days |
| Follicular phase | 9.53 | ± | 1.60 | days |
| Preovulatory phase | 15.63 | ± | 3.98 | days |
| Mid-luteal phase | 24.63 | ± | 3.95 | days |
| Length between mid-luteal phase and the next menstruation | 6.39 | ± | 2.89 | days |
| Number of menstrual cycles required to complete the experiment | 7.16 | ± | 2.91 | cycles |

Table S2. Psychological raw data in each menstrual cycle phase

|  | MP | | | FP | | | OP | | | LP | | | |
| --- | --- | --- | --- | --- | --- | --- | --- | --- | --- | --- | --- | --- | --- |
| MDQ (points) |  |  |  |  |  |  |  |  |  |  |  |  |  |
| Pain | 8.21 | ± | 6.57 | 6.32 | ± | 5.65 | 4.89 | ± | 4.38 | 5.21 | ± | 4.55 |  |
| Behavioral change | 7.95 | ± | 6.75 | 7.37 | ± | 6.89 | 5.79 | ± | 5.92 | 6.84 | ± | 6.01 |  |
| Water retention | 3.89 | ± | 3.86 | 2.84 | ± | 3.72 | 1.84 | ± | 1.81 | 2.63 | ± | 2.81 |  |
| Arousal | 2.42 | ± | 3.84 | 2.63 | ± | 3.70 | 2.58 | ± | 3.87 | 2.05 | ± | 3.63 |  |
| Concentration | 7.95 | ± | 10.10 | 7.16 | ± | 8.76 | 4.53 | ± | 6.24 | 6.21 | ± | 8.19 |  |
| Autonomic reactions | 2.00 | ± | 2.94 | 1.37 | ± | 2.13 | 0.42 | ± | 1.18 | 1.26 | ± | 2.24 |  |
| Negative affect | 8.63 | ± | 11.03 | 9.58 | ± | 11.55 | 5.47 | ± | 8.17 | 7.47 | ± | 10.71 |  |
| Control | 1.05 | ± | 1.50 | 1.26 | ± | 2.55 | 0.68 | ± | 1.26 | 1.42 | ± | 2.48 |  |
| Total scores | 42.11 | ± | 42.89 | 38.53 | ± | 40.12 | 26.21 | ± | 28.01 | 33.11 | ± | 36.88 |  |

Mean±SD. MP, menstruation phase; FP, follicular phase; OP, preovulatory phase; LP, mid-luteal phase; MDQ, mental distress questionnaire.

Table S3. Neurophysiological raw data in each menstrual cycle phase

|  | | MP | | | FP | | | OP | | | | LP | | | |
| --- | --- | --- | --- | --- | --- | --- | --- | --- | --- | --- | --- | --- | --- | --- | --- |
| N20 | |  |  |  |  |  |  |  |  |  |  | |  |  |  |
|  | Latency (ms) | 18.14 | ± | 0.76 | 18.16 | ± | 0.81 | 18.19 | ± | 0.69 | 17.98 | | ± | 0.63 |  |
|  | Amplitude (µV) | -3.08 | ± | 1.07 | -3.15 | ± | 1.27 | -3.01 | ± | 1.27 | -3.11 | | ± | 1.31 |  |
| RMT | | 51.59 | ± | 7.52 | 53.71 | ± | 8.19 | 52.47 | ± | 7.90 | 52.65 | | ± | 8.97 |  |
| AMT | | 38.29 | ± | 5.94 | 38.94 | ± | 4.68 | 37.88 | ± | 4.64 | 37.00 | | ± | 4.27 |  |
| TS | | 64.88 | ± | 10.98 | 67.82 | ± | 10.96 | 65.29 | ± | 9.80 | 65.29 | | ± | 12.47 |  |
| 100%_MEP (µV) | | 0.10 | ± | 0.07 | 0.12 | ± | 0.08 | 0.16 | ± | 0.12 | 0.18 | | ± | 0.14 |  |
| 110%_MEP (µV) | | 0.52 | ± | 0.28 | 0.52 | ± | 0.37 | 0.57 | ± | 0.22 | 0.60 | | ± | 0.24 |  |
| 120%_MEP (µV) | | 1.06 | ± | 0.60 | 1.00 | ± | 0.63 | 1.12 | ± | 0.42 | 1.16 | | ± | 0.71 |  |
| 130%_MEP (µV) | | 1.54 | ± | 0.83 | 1.45 | ± | 0.71 | 1.64 | ± | 0.74 | 1.81 | | ± | 0.98 |  |
| 140%_MEP (µV) | | 2.04 | ± | 1.15 | 1.82 | ± | 0.95 | 1.98 | ± | 0.76 | 2.16 | | ± | 1.23 |  |
| 150%_MEP (µV) | | 2.28 | ± | 1.33 | 2.42 | ± | 1.29 | 2.28 | ± | 1.02 | 2.46 | | ± | 1.29 |  |
| Unconditioned MEP (µV) | | 1.08 | ± | 0.08 | 1.08 | ± | 0.12 | 1.09 | ± | 0.09 | 1.07 | | ± | 0.08 |  |
| SICI (µV) | | 0.63 | ± | 0.30 | 0.64 | ± | 0.21 | 0.68 | ± | 0.26 | 0.69 | | ± | 0.31 |  |
| SICF (µV) | | 1.51 | ± | 0.45 | 1.67 | ± | 0.85 | 1.70 | ± | 0.61 | 1.69 | | ± | 0.71 |  |
| SAI_2ms (µV) | | 0.69 | ± | 0.31 | 0.64 | ± | 0.29 | 0.78 | ± | 0.27 | 0.57 | | ± | 0.22 |  |
| SAI_10ms (µV) | | 1.17 | ± | 0.31 | 1.05 | ± | 0.25 | 1.24 | ± | 0.33 | 1.28 | | ± | 0.46 |  |

Mean±SD. MP, menstruation phase; FP, follicular phase; OP, preovulatory phase; LP, mid-luteal phase; RMT, resting motor threshold; AMT, active motor threshold; TS, test stimulation; MEP, motor-evoked potential; SICI, short-interval intracortical inhibition; SICF, short-interval intracortical facilitation; SAI, short-latency afferent inhibition.

Table S4. The raw data of statistical analysis using glmer (fixed effects: cycle phase)

| E2 | Estimate Std | Error | t value | Pr (>\|z\|) |
| --- | --- | --- | --- | --- |
| (intercept) | 0.81063 | 0.10673 | 7.595 | 3.08e-14 |
| phase[T.lut] | 0.18521 | 0.08683 | 2.133 | 0.03293 |
| phase[T.mense] | 0.05932 | 0.08683 | 0.683 | 0.49455 |
| phase[T.ovul] | 0.28968 | 0.08683 | 3.336 | 0.00085 |
|  |  |  |  |  |
| (intercept) | 1.10032 | 0.10673 | 10.309 | < 2e-16 |
| phase[T.folli] | -0.28968 | 0.08683 | -3.336 | 0.00085 |
| phase[T.lut] | -0.10447 | 0.08683 | -1.203 | 0.22892 |
| phase[T.mense] | -0.23037 | 0.08683 | -2.653 | 0.00798 |
|  |  |  |  |  |
| (intercept) | 0.99584 | 0.10673 | 9.330 | < 2e-16 |
| phase[T.folli] | -0.18521 | 0.08683 | -2.133 | 0.0329 |
| phase[T.mense] | -0.12589 | 0.08683 | -1.450 | 0.1471 |
| phase[T.ovul] | 0.10447 | 0.08683 | 1.203 | 0.2289 |
|  |  |  |  |  |
| P4 | Estimate Std | Error | t value | Pr (>\|z\|) |
| (intercept) | 90.086 | 19.124 | 4.711 | 0.000002468 |
| phase[T.lut] | 109.205 | 20.646 | 5.289 | 0.000000123 |
| phase[T.mense] | -17.886 | 20.646 | -0.866 | 0.386 |
| phase[T.ovul] | 2.327 | 20.646 | 0.113 | 0.910 |
|  |  |  |  |  |
| (intercept) | 92.413 | 19.124 | 4.832 | 0.000001349 |
| phase[T.folli] | -2.327 | 20.646 | -0.113 | 0.910 |
| phase[T.lut] | 106.878 | 20.646 | 5.177 | 0.000000226 |
| phase[T.mense] | -20.213 | 20.646 | -0.979 | 0.328 |
|  |  |  |  |  |
| (intercept) | 199.29 | 19.12 | 10.421 | < 2e-16 |
| phase[T.folli] | -109.21 | 20.65 | -5.289 | 1.23e-07 |
| phase[T.mense] | -127.09 | 20.65 | -6.156 | 7.47e-10 |
| phase[T.ovul] | -106.88 | 20.65 | -5.177 | 2.26e-07 |
|  |  |  |  |  |
| MDQ score | Estimate Std | Error | z value | Pr (>\|z\|) |
| (intercept) | 3.09937 | 0.26210 | 11.825 | < 2e-16 |
| phase[T.lut] | -0.15165 | 0.05427 | -2.794 | 0.0052 |
| phase[T.mense] | 0.08883 | 0.05106 | 1.740 | 0.0819 |
| phase[T.ovul] | -0.38518 | 0.05799 | -6.643 | 3.08e-11 |
|  |  |  |  |  |
| (intercept) | 2.71418 | 0.26332 | 10.308 | < 2e-16 |
| phase[T.folli] | 0.38518 | 0.05799 | 6.643 | 3.08e-11 |
| phase[T.lut] | 0.23353 | 0.05988 | 3.900 | 9.62e-05 |
| phase[T.mense] | 0.47401 | 0.05698 | 8.319 | < 2e-16 |
|  |  |  |  |  |
| (intercept) | 2.94773 | 0.26253 | 11.228 | < 2e-16 |
| phase[T.folli] | 0.15165 | 0.05427 | 2.794 | 0.0052 |
| phase[T.mense] | 0.24048 | 0.05320 | 4.520 | 0.00000617 |
| phase[T.ovul] | -0.23353 | 0.05988 | -3.900 | 0.00009616 |
|  |  |  |  |  |
| GPT score | Estimate Std | Error | t value | Pr (>\|z\|) |
| (intercept) | 53.817895 | 1.279137 | 42.074 | < 2e-16 |
| phase[T.lut] | -1.202632 | 1.051533 | -1.144 | 0.253 |
| phase[T.mense] | 0.003684 | 1.051532 | 0.004 | 0.997 |
| phase[T.ovul] | -0.728421 | 1.051531 | -0.693 | 0.488 |
|  |  |  |  |  |
| FMT score | Estimate Std | Error | z value | Pr (>\|z\|) |
| (intercept) | 13.5708966 | 0.2294159 | 59.15 | < 2e-16 |
| phase[T.lut] | -0.0590098 | 0.0003709 | -159.12 | < 2e-16 |
| phase[T.mense] | -0.0993472 | 0.0003748 | -265.09 | < 2e-16 |
| phase[T.ovul] | -0.0969816 | 0.0003745 | -258.94 | < 2e-16 |
|  |  |  |  |  |
| IO curve inclination | Estimate Std | Error | z value | Pr (>\|z\|) |
| (intercept) | -0.85871 | 0.43906 | -1.956 | 0.0505 |
| phase[T.lut] | 0.07369 | 0.50413 | 0.146 | 0.8838 |
| phase[T.mense] | 0.02138 | 0.51061 | 0.042 | 0.9666 |
| phase[T.ovul] | -0.01460 | 0.51521 | -0.028 | 0.9774 |
|  |  |  |  |  |
| SICI | Estimate Std | Error | t value | Pr (>\|z\|) |
| (intercept) | 59.995 | 5.941 | 10.098 | < 2e-16 |
| phase[T.lut] | 3.989 | 4.710 | 0.847 | 0.397 |
| phase[T.mense] | -1.271 | 4.710 | -0.270 | 0.787 |
| phase[T.ovul] | 2.301 | 4.710 | 0.488 | 0.625 |
|  |  |  |  |  |
| SICF | Estimate Std | Error | t value | Pr (>\|z\|) |
| (intercept) | 156.5862 | 14.7186 | 10.639 | < 2e-16 |
| phase[T.lut] | 2.9880 | 10.5478 | 0.283 | 0.777 |
| phase[T.mense] | -17.2105 | 10.5444 | -1.632 | 0.103 |
| phase[T.ovul] | -0.5132 | 10.5478 | -0.049 | 0.961 |
|  |  |  |  |  |
| SAI_2ms | Estimate Std | Error | z value | Pr (>\|z\|) |
| (intercept) | 58.860 | 1.876 | 31.371 | < 2e-16 |
| phase[T.lut] | -4.755 | 2.577 | -1.845 | 0.065 |
| phase[T.mense] | 4.796 | 2.684 | 1.787 | 0.074 |
| phase[T.ovul] | 11.943 | 2.761 | 4.325 | 0.0000153 |
|  |  |  |  |  |
| (intercept) | 70.804 | 2.055 | 34.454 | < 2e-16 |
| phase[T.folli] | -11.943 | 2.761 | -4.325 | 1.53e-05 |
| phase[T.lut] | -16.699 | 2.710 | -6.161 | 7.22e-10 |
| phase[T.mense] | -7.148 | 2.812 | -2.542 | 0.011 |
|  |  |  |  |  |
| (intercept) | 54.105 | 1.800 | 30.056 | < 2e-16 |
| phase[T.folli] | 4.755 | 2.577 | 1.845 | 0.065037 |
| phase[T.mense] | 9.551 | 2.632 | 3.629 | 0.000284 |
| phase[T.ovul] | 16.699 | 2.710 | 6.161 | 7.22e-10 |
|  |  |  |  |  |
| SAI_10ms | Estimate Std | Error | z value | Pr (>\|z\|) |
| (intercept) | 96.979 | 2.401 | 40.397 | < 2e-16 |
| phase[T.lut] | 22.426 | 3.568 | 6.268 | 3.25e-10 |
| phase[T.mense] | 11.857 | 3.479 | 3.408 | 0.000655 |
| phase[T.ovul] | 17.582 | 3.527 | 4.984 | 6.21e-07 |
|  |  |  |  |  |
| (intercept) | 119.405 | 2.661 | 44.868 | < 2e-16 |
| phase[T.folli] | -22.426 | 3.568 | -6.286 | 3.25e-10 |
| phase[T.mense] | -10.569 | 3.664 | -2.885 | 0.00392 |
| phase[T.ovul] | -4.844 | 3.710 | -1.306 | 0.19163 |

Mean±SD. mense, menstruation phase; folli, follicular phase; ovul, preovulatory phase; lut, mid-luteal phase; MDQ, mental distress questionnaire; GPT, grooved pegboard task; FMT, force modulation task; IO curve inclination, input-output curve; SICI, short-interval intracortical inhibition; SICF, short-interval intracortical facilitation; SAI, short-latency afferent inhibition.

Table S5. The relationship between E2 level and behavioral and neurophysiological data

|  | r | 95% CI | p |
| --- | --- | --- | --- |
| MDQ score | 0.09 | [-0.17, 0.34] | 0.48 |
| GPT score | -0.14 | [-0.38, 0.13] | 0.31 |
| FMT score | 0.06 | [-0.20, 0.31] | 0.66 |
| IO curve inclination | -0.12 | [-0.38, 0.16] | 0.41 |
| SICI | -0.03 | [-0.30, 0.24] | 0.81 |
| SICF | 0.01 | [-0.26, 0.28] | 0.93 |
| SAI_2ms | -0.01 | [-0.28, 0.26] | 0.93 |
| SAI_10ms | 0.13 | [-0.15, 0.39] | 0.36 |


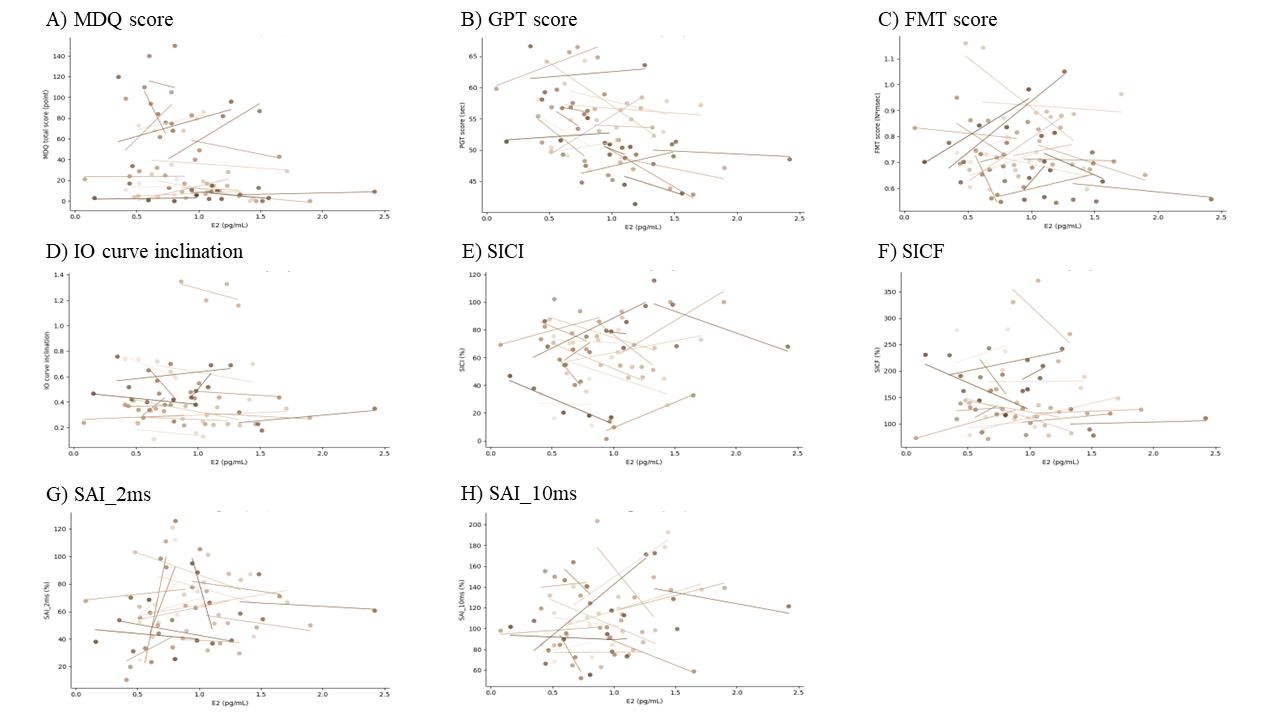
E2, estradiol; 95% CI, 95% confidence interval; MDQ, mental distress questionnaire; GPT, grooved pegboard task; FMT, force modulation task; IO curve inclination, input-output curve; SICI, short-interval intracortical inhibition; SICF, short-interval intracortical facilitation; SAI, short-latency afferent inhibition.

Figure S1. The relationship between E2 level and behavioral and neurophysiological data

Table S6. The relationship between P4 level and behavioral and neurophysiological data

|  | r | 95% CI | p |
| --- | --- | --- | --- |
| MDQ score | 0.14 | [-0.12, 0.38] | 0.29 |
| GPT score | -0.11 | [-0.36, 0.15] | 0.40 |
| FMT score | 0.14 | [-0.12, 0.39] | 0.28 |
| IO curve inclination | 0.08 | [-0.20, 0.35] | 0.57 |
| SICI | 0.07 | [-0.21, 0.34] | 0.63 |
| SICF | 0.06 | [-0.22, 0.33] | 0.68 |
| SAI_2ms | -0.19 | [-0.45, 0.08] | 0.16 |
| SAI_10ms | 0.21 | [-0.07, 0.46] | 0.14 |


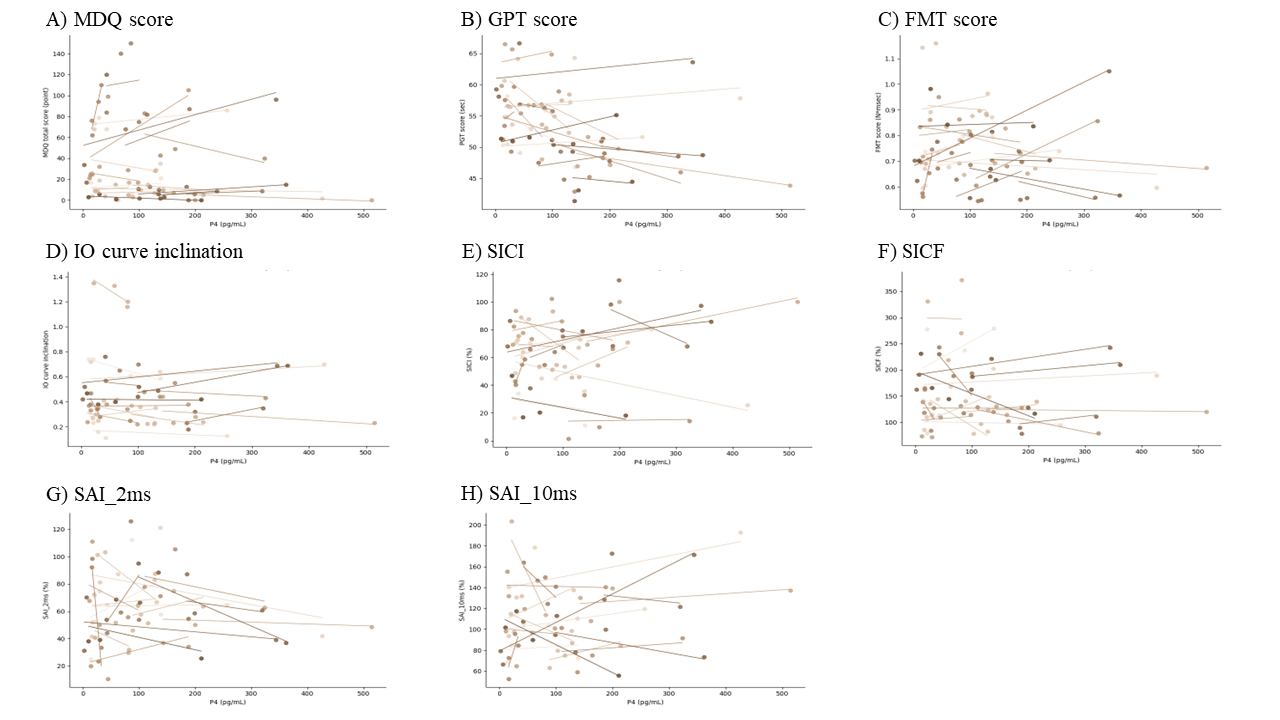
P4, progesterone; 95% CI, 95% confidence interval; MDQ, mental distress questionnaire; GPT, grooved pegboard task; FMT, force modulation task; IO curve inclination, input-output curve; SICI, short-interval intracortical inhibition; SICF, short-interval intracortical facilitation; SAI, short-latency afferent inhibition.

Figure S2. The relationship between P4 level and behavioral and neurophysiological data
